# Development and evaluation of a method for tumor growth simulation in virtual clinical trials of breast cancer screening

**DOI:** 10.1117/1.JMI.9.3.033503

**Published:** 2022-06-06

**Authors:** Hanna Tomic, Anna Bjerkén, Gustav Hellgren, Kristin Johnson, Daniel Förnvik, Sophia Zackrisson, Anders Tingberg, Magnus Dustler, Predrag R. Bakic

**Affiliations:** a Lund University, Medical Radiation Physics, Department of Translational Medicine, Malmö, Sweden; b Skåne University Hospital, Radiation Physics, Malmö, Sweden; c Lund University, Diagnostic Radiology, Department of Translational Medicine, Malmö, Sweden; d Skåne University Hospital, Department of Medical Imaging and Physiology, Malmö, Sweden; e University of Pennsylvania, Department of Radiology, Philadelphia, Pennsylvania, United States

**Keywords:** digital mammography, tumor growth, tumor volume doubling time, simulation, virtual clinical trials

## Abstract

**Purpose:**

Image-based analysis of breast tumor growth rate may optimize breast cancer screening and diagnosis by suggesting optimal screening intervals and guide the clinical discussion regarding personalized screening based on tumor aggressiveness. Simulation-based virtual clinical trials (VCTs) can be used to evaluate and optimize medical imaging systems and design clinical trials. This study aimed to simulate tumor growth over multiple screening rounds.

**Approach:**

This study evaluates a preliminary method for simulating tumor growth. Clinical data on tumor volume doubling time (TVDT) was used to fit a probability distribution (“clinical fit”) of TVDTs. Simulated tumors with TVDTs sampled from the clinical fit were inserted into 30 virtual breasts (“simulated cohort”) and used to simulate mammograms. Based on the TVDT, two successive screening rounds were simulated for each virtual breast. TVDTs from clinical and simulated mammograms were compared. Tumor sizes in the simulated mammograms were measured by a radiologist in three repeated sessions to estimate TVDT.

**Results:**

The mean TVDT was 297 days (standard deviation, SD, 169 days) in the clinical fit and 322 days (SD, 217 days) in the simulated cohort. The mean estimated TVDT was 340 days (SD, 287 days). No significant difference was found between the estimated TVDTs from simulated mammograms and clinical TVDT values (p>0.5). No significant difference (p>0.05) was observed in the reproducibility of the tumor size measurements between the two screening rounds.

**Conclusions:**

The proposed method for tumor growth simulation has demonstrated close agreement with clinical results, supporting potential use in VCTs of temporal breast imaging.

## 1 Introduction

Breast cancer screening programs based on x-ray imaging have demonstrated the ability to reduce cancer mortality and improve treatment results.^1^ These programs are challenged by undetected cancers, overdiagnosis, and overtreatment.^2^^,^^3^ New alternative clinical imaging modalities, such as digital breast tomosynthesis (DBT), have resulted in improved cancer detection rates, with mixed effects on recall rates reported by different clinical trials.^4^

Clinical trials of medical imaging systems are time-consuming, expensive, and depend on patient inclusion (i.e., finding a sufficient number of patients with pathologies that have been anticipated during the study design). A possible preclinical alternative to conventional clinical trials is virtual clinical trials (VCTs) based on computer modeling. VCTs have been used to validate pharmaceuticals, medical devices, and therapeutic or diagnostic tools.^5^^–^^8^ The advantages of VCTs include the possibility of performing multiple and/or repeated studies for comparing, optimizing, and assessing reproducibility without being dependent on available patients or exposing them to the risks that may come from *in vivo* studies. Another benefit of VCTs is the high level of control over the study parameters, such as the characteristics of the virtual patients or medical devices. This allows focused studies, which would be prohibitive to perform clinically. In this paper, we focused on VCTs for breast cancer screening.^6^^,^^8^

There are several simulation platforms available for designing and performing VCTs of breast imaging.^9^^–^^11^ These platforms provide models of breast anatomy, image acquisition, and image interpretation. This study focuses on OpenVCT, an open-source platform developed at the University of Pennsylvania.^10^ OpenVCT has been shown to successfully predict the outcome of clinical trials of breast cancer screening using digital mammography (DM) and DBT.^12^ To date, the growth and progression of breast tumors have not been simulated in OpenVCT.

This work aimed to expand the capabilities of VCTs for breast cancer screening. Specifically, the aim was to develop a method for simulating tumor growth in virtual lesions based on clinical data for the purpose of simulating consecutive mammograms. This would allow simulation of breast cancer screening in the same patient population over multiple rounds, improving the realism of the simulated trials. Our motivation was to design a virtual continuation of our recently completed clinical trial of DBT breast cancer screening, the Malmö Breast Tomosynthesis Screening Trial (MBTST),^13^ one of the largest prospective European DBT screening trials.

A recent review shows that many studies have adopted the concept of tumor volume doubling time (TVDT) to quantify tumor growth in breast cancer.^14^ In breast cancer research, TVDT has been used for disease-specific prediction models and optimization of screening programs.^14^^,^^15^ However, TVDT has not been generally accepted in everyday clinical practice as an addition to conventional tumor grading. This may be related to the lack of standardized methods for estimating tumor growth rate. Yet, the clinical potential of tumor growth analysis is supported by reports that TVDT values of different breast cancer subtypes differ significantly.^16^ Based upon the reported clinical relevance, our project would enable the simulation of tumor growth, to investigate and optimize the practical use of TVDT analysis.

As for any biological process described by mathematics, TVDT also comes with its limitations and simplifications. Previous studies, reviewed by Dahan et al.,^14^ of tumor growth in terms of TVDT have frequently used exponential growth functions and spherical tumor approximation. Another approach to model tumor growth is by the Gompertz function. The Gompertz function describes the growth rate as sigmoidal, meaning that it has a more lethargic onset and terminates in a plateau. The Gompertz function could potentially be used to depict the transition in tumor growth rate when the tumor reaches a certain size.^17^ However, for short measurement intervals, the exponential function has been found to work well.^14^^,^^18^ Studies have also shown no significant difference between using the Gompertz growth function and the exponential function.^19^ Moreover, it has been hypothesized that the exponential function is most likely an accurate growth model representation for typical tumor sizes seen in mammography screening (5 to 35 mm).^19^ As a result, we decided to adopt the exponential growth function in our study.

When it comes to the tumor shape approximation, it is not commonly accepted how to measure the tumor extent on mammography, that is to include or not include the spiculations.^20^ Most radiologists report the bulk size (making the spiculations superfluous). For the purpose of estimating TVDT, the spherical shape provides more practical control of study conditions. Our preliminary study focused on the effects of tumor size and growth rate; therefore, we have simulated spherical tumors.

Studies regarding simulations of breast tumor growth in mammographic projections have been quite limited. Various mathematical methods for simulating breast tumors have been reported,^21^^–^^26^ but they have not simulated tumor progression over time. On the other hand, changes in tumor diameter or volume have been modeled,^27^^–^^29^ without simulated tumor appearance. Recently, Sengupta et al.^30^ simulated tumors of breast-like morphology at different points in time. The reported studies have not simulated mammographic projections from a cohort of virtual patients. Our goal is to simulate tumor appearance in mammographic projections at different screening time points, with tumor growth rates and TVDT distribution matching the clinical results. This would increase the confidence in virtual trials over multiple screening rounds.

## 2 Materials and Methods

### 2.1 Theoretical Analysis of the Tumor Growth Rate

**Fig. 1:**
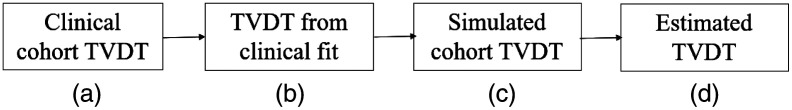
TVDT values used in the analysis. Clinical TVDTs from our previous clinical study (a) were used to obtain a clinically fitted probability distribution (b). A selection of 30 doubling times was randomly sampled from the clinical fit and used to simulate growing tumors in virtual mammograms of the simulated cohort (c). A radiologist manually segmented tumors from the simulated cohort to estimate TVDTs (d) in three repeated measurement sessions.

The tumor growth model in this study was based on our previous analysis of clinical TVDT estimated from 31 breast cancer patients imaged at Skåne University Hospital in Malmö, Sweden [Fig. 1(a)].^19^ To generalize the clinical data, a gamma probability distribution was fitted to the histogram of clinical TVDT values [Fig. 1(b)]. These values have been referred to as “clinical fit” and were used to simulate consecutive mammograms with growing tumors as detailed below.

The clinical fit was randomly sampled to select 30 virtual patients and their corresponding TVDT values to obtain the simulated cohort [Fig. 1(c)]. In the last step, a radiologist manually segmented tumors from the simulated cohort using three repeated measurement sessions. Estimated TVDT values [Fig. 1(d)] were calculated based on each of these measurements.

The tumor growth model was assumed to follow an exponential function and the tumors were approximated as spherical; thus, the tumor size, d, could be inferred at any time point, t, based on the individual TVDT

$$\;\mathrm{TVDT}\;=\frac{\ln\;2\cdot \Delta t}{3\cdot (\ln\;d_{1}-\ln\;d_{2})}.$$ (1)

To evaluate the tumor growth model, we simulated two mammograms for each virtual patient, one for each of the two consecutive screening time points. At each time point, the tumor size was inferred from Eq. (1). The time interval between the simulated prior mammogram (“first screening”) and the simulated later mammogram (“second screening”) was set to either 18 or 24 months based on the screening program at our institution: 18 months for women 40 to 54 years of age and 24 months for women 55 to 74 years.^31^

### 2.2 Simulation Technique

OpenVCT, open-source software that simulates breast anatomy, mammographic imaging, and image interpretation for the purpose of performing VCTs,^10^ was used for this study. The software enables the insertion of simulated tumors and the generation of mammographic images of a simulated cohort of women. Each tumor was approximated by a sphere that corresponded to a radiographic mass in the simulated mammogram. The tumor diameter in the simulated first screening mammogram was randomly selected from 3.5 to 13 mm, representing sizes just below the reported range of clinically detectable lesions in mammograms (7.5 to 15 mm).^19^^,^^32^^–^^34^ This was done to achieve measurable sizes in the first (prior) screening mammogram and clinically relevant sizes in the second (later) screening mammogram. The tumor location was chosen randomly throughout the simulated fat and dense breast tissue regions. The patient age was selected based on the age at detection of 139 breast cancers from the MBTST study.^13^ Out of the 139 cancer cases in MBTST, 110 were spiculated or circumscribed masses. Simulated breast volumes were 450–950 mL. Breast density was selected randomly in the OpenVCT user interface, where it is defined as the number of dense compartments in the simulated breast (5% to 60%).

The mammographic projections were simulated by imaging geometry and radiographic techniques corresponding to a clinical imaging system (Selenia Dimensions, Hologic, Inc., Bedford, Massachusetts, United States). The simulated acquisition system has a spatial resolution of 70  μm and a source-to-detector distance of 700 mm. The simulated radiographic projections were processed using a commercially available software library (Briona Standard, Real Time Tomography, LLC, Villanova, Pennsylvania, United States) before being used in the observer study.

### 2.3 Observer Study

**Fig. 2:**
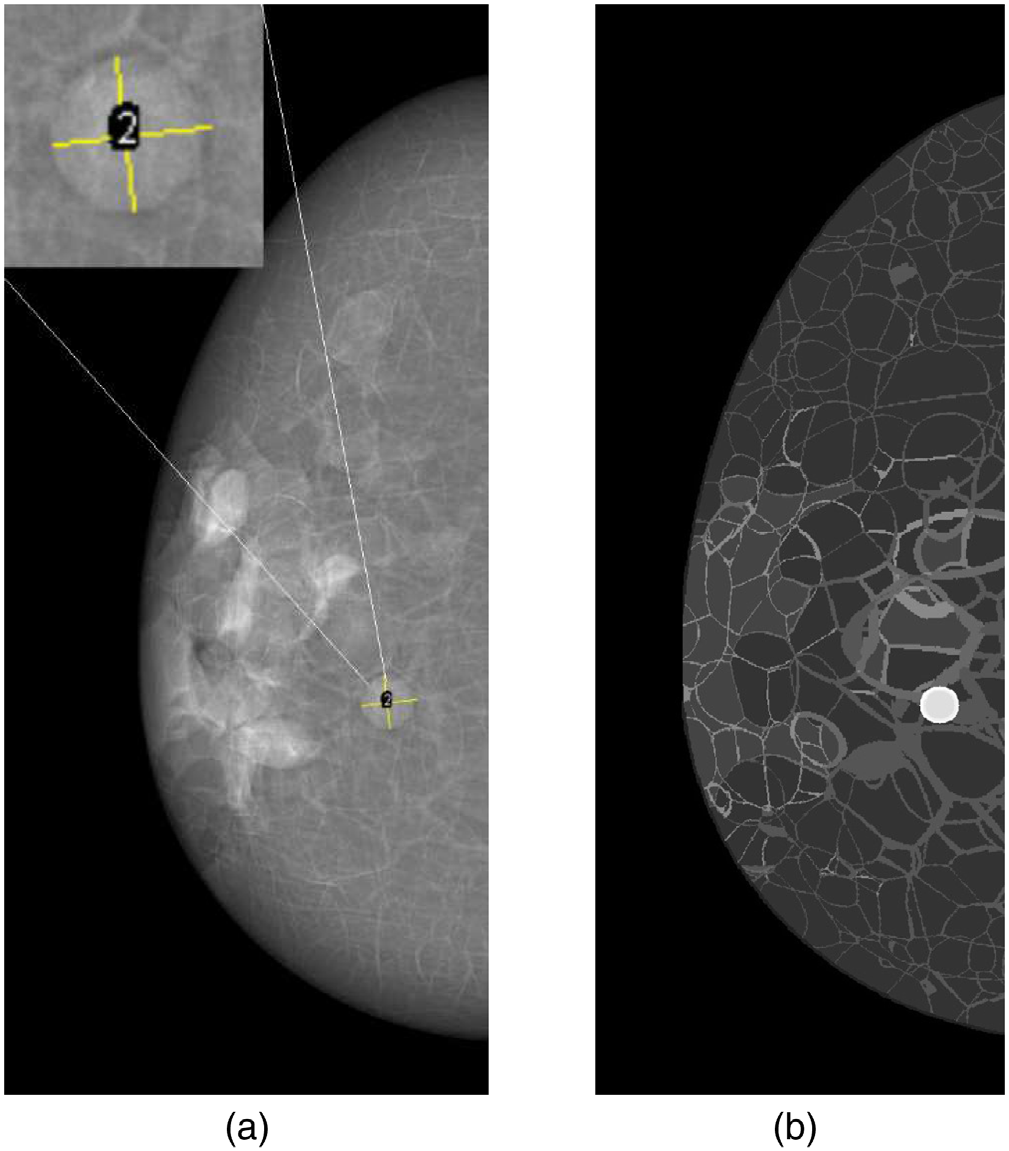
Tumor size measured in simulated mammograms for evaluation of the tumor growth simulation method (a). Two sizes in approximately orthogonal directions (yellow lines in the insert) were measured by a radiologist. An average of the two measured sizes was used to estimate the tumor volume. The tumor can also be seen as the bright spot in the cross-section of the phantom (b). The light gray compartments in the breast phantom consist of fibroglandular tissue, whilst the darker gray is adipose tissue (fat).

A human reader study was performed to estimate TVDTs from simulated mammograms. Simulated mammograms were reviewed by one radiologist (KJ). The radiologist was asked to measure the largest tumor size and the size in the direction approximately orthogonal to the largest size (Fig. 2). The size was recorded with 0.1-mm precision, higher than reporting tumor size in daily clinical practice. The radiologist was informed of the tumor location since the study was not intended as a search task. However, the margin was to be determined by the radiologist, as a part of the tumor size measurement. The tumor volume was estimated from a spherical approximation using the mean of the two measured orthogonal tumor sizes. The measurements were repeated in three sessions. The first and second measurement sessions were separated by seven months, and the second and third measurements by 1.5 months. For each session, the images were displayed on a standard high-resolution, high-contrast display (Lenovo Thinkvision P24h-10). The radiologist used ImageJ software (National Institutes of Health, Bethesda, Maryland, United States) to manually indicate the tumor size. The mean of the two measured tumor sizes was used in Eq. (1) to estimate the TVDT.

### 2.4 Statistical Analysis and Comparison to Clinical Data

The tumor size measurements and analyzed TVDT data sets were tested for normality using the Shapiro–Wilk test.^35^ The reproducibility between the three tumor size measurement sessions was assessed by the Friedman test.^36^ Each TVDT data set was assigned a probability distribution based on the Anderson–Darling (AD) goodness-of-fit test.^37^

The estimated TVDTs [Fig. 1(d)] were compared with the TVDT values from the simulated cohort [Fig. 1(c)] and the clinical data [Fig. 1(a)]. In addition, the clinical fit [Fig. 1(b)] was compared with the clinical TVDT values [Fig. 1(a)] and the TVDT values from the simulated cohort [Fig. 1(c)]. The comparison was performed using the two-sample Kolmogorov–Smirnov test and the Wilcoxon signed-rank test, which are suitable even for non-normal distributions.^38^^–^^40^

The absolute error between simulated and estimated TVDTs was compared with the simulated tumor sizes to assess whether tumor size affects the TVDT estimation.

For the purpose of our analysis, the tumor cases were also divided into fast- and slow-growing tumors. They were defined as tumors belonging to either the lowest or highest quartile (25%) of the simulated TVDT data set. The relationship between simulated (the nominal TVDT values) and estimated TVDTs was compared between fast- and slow-growing tumors using the two-sample t-test. All statistical tests were performed at a 0.05 significance level.

## 3 Results

**Fig. 3:**
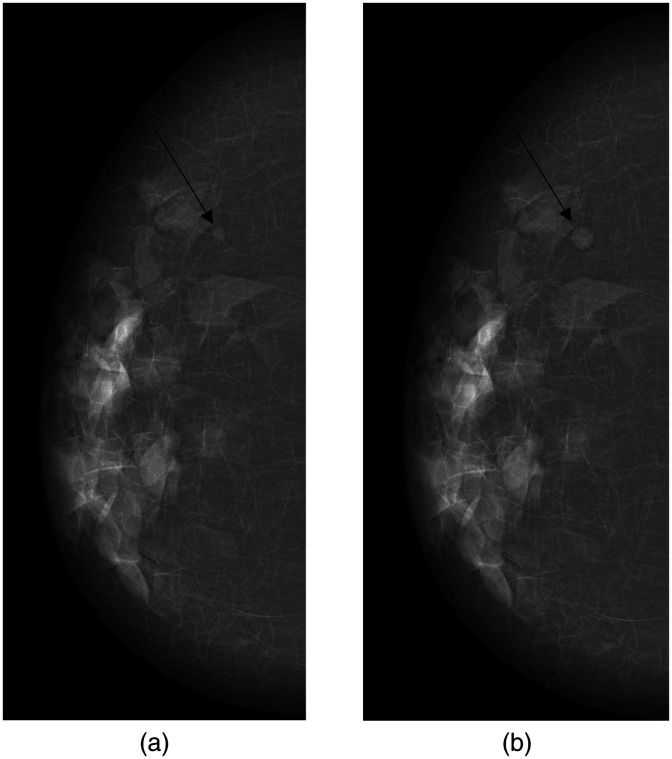
Two simulated mammograms of the same breast in a virtual patient. The simulated mammograms correspond to a 700 mL virtual breast (approx. C-cup) with 15% dense compartments. It has a ligament thickness of 0.02 cm and a skin thickness of 0.15 cm. The patient was assigned a TVDT of 317 days. (a) The tumor at the first virtual screening occasion was 4.0 mm. (b) The tumor after 24 months had grown to 6.8 mm. The radiologist estimated the sizes to be 4.1 and 7.3 mm, respectively. The estimated doubling time was 294 days.

**Fig. 4:**
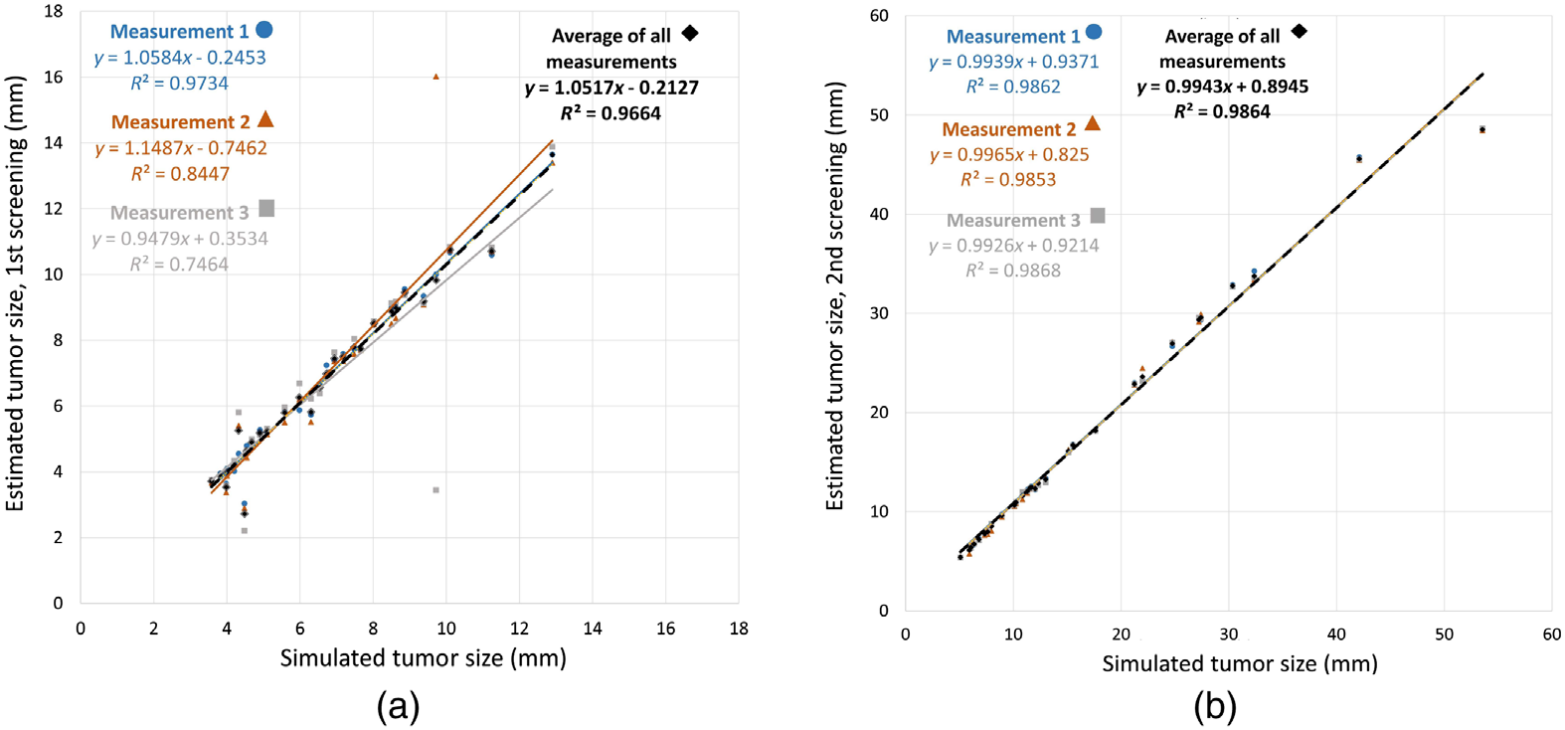
The tumor size simulated in the first (a) and second (b) screening round (18/24 months after the first one) was compared with the size measured by the radiologist in three measurement sessions. The linear regression is also shown.

Figure 3 shows an example of mammograms with a tumor simulated at two consecutive screening time points separated by 24 months. The relationship between the simulated and estimated tumor size is shown for the first (prior) simulated screening round in Fig. 4(a) and the second (later) simulated screening in Fig. 4(b).

Based on the R-squared values, the data points are closer to the fitted regression line in the second screening round [Fig. 4(b)] compared with the first screening round [Fig. 4(a)]. The Friedman test yielded the p-values of the difference between the three repeated size measurements in the first (p=0.33) and second (p=0.83) simulated screening rounds.

**Fig. 5:**
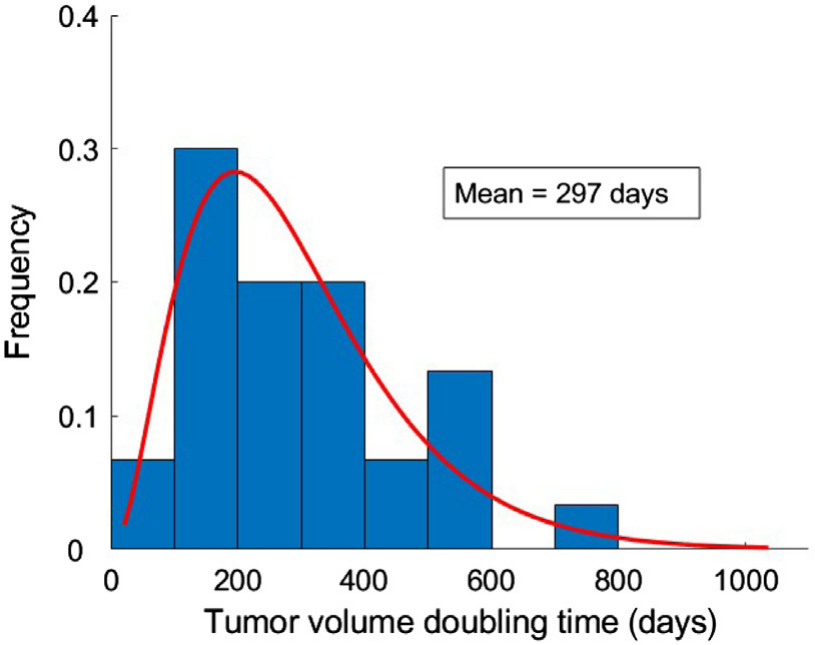
Histogram of TVDT values from the clinical cohort of 31 women with breast cancer from our previous study.^19^ The red line represents the fitted distribution (“clinical fit”).

**Fig. 6:**
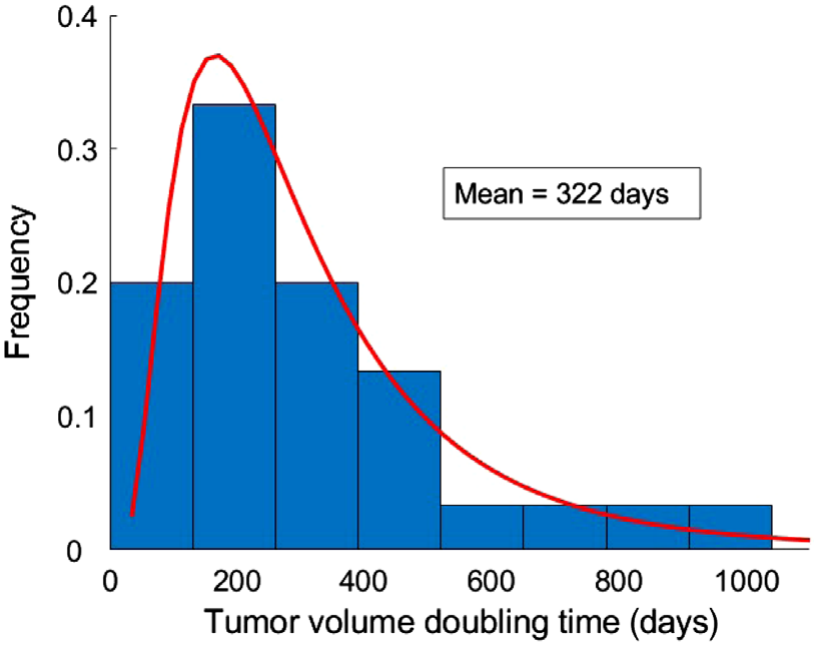
Histogram of TVDT values from the simulated cohort of 30 breast tumors. The values were sampled from the gamma distribution fit in Fig. 5. The red line represents the distribution fitted to the simulated cohort.

**Fig. 7:**
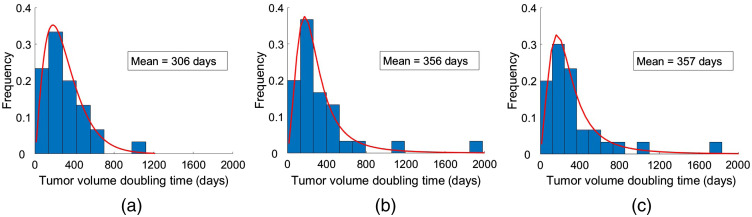
Histograms of TVDT values estimated by a radiologist in the (a) first, (b) second, and (c) third tumor size measurement sessions. The red lines represent their corresponding distribution fits.

The clinical data reported in our previous study had a mean TVDT of 282 days (standard deviation, SD, 164 days) (Fig. 5).^19^ The clinical fit had a mean TVDT of 297 (SD, 169 days), whereas the simulated patient cohort had 322 days (SD, 217 days) (Fig. 6). The estimated mean TVDTs for the simulated mammograms were 306 days (SD, 209 days), 356 days (SD, 369 days), and 357 days (SD, 344 days) for the three tumor size measurement sessions, respectively (Fig. 7). The average estimated TVDT was 340 days (SD, 287 days).

The clinical, simulated, and estimated TVDT histograms and probability distributions (Figs. 5–7) are skewed toward TVDTs of ∼200  days, with a characteristic tail that takes into account slow-growing tumors with longer TVDTs.

**Fig. 8:**
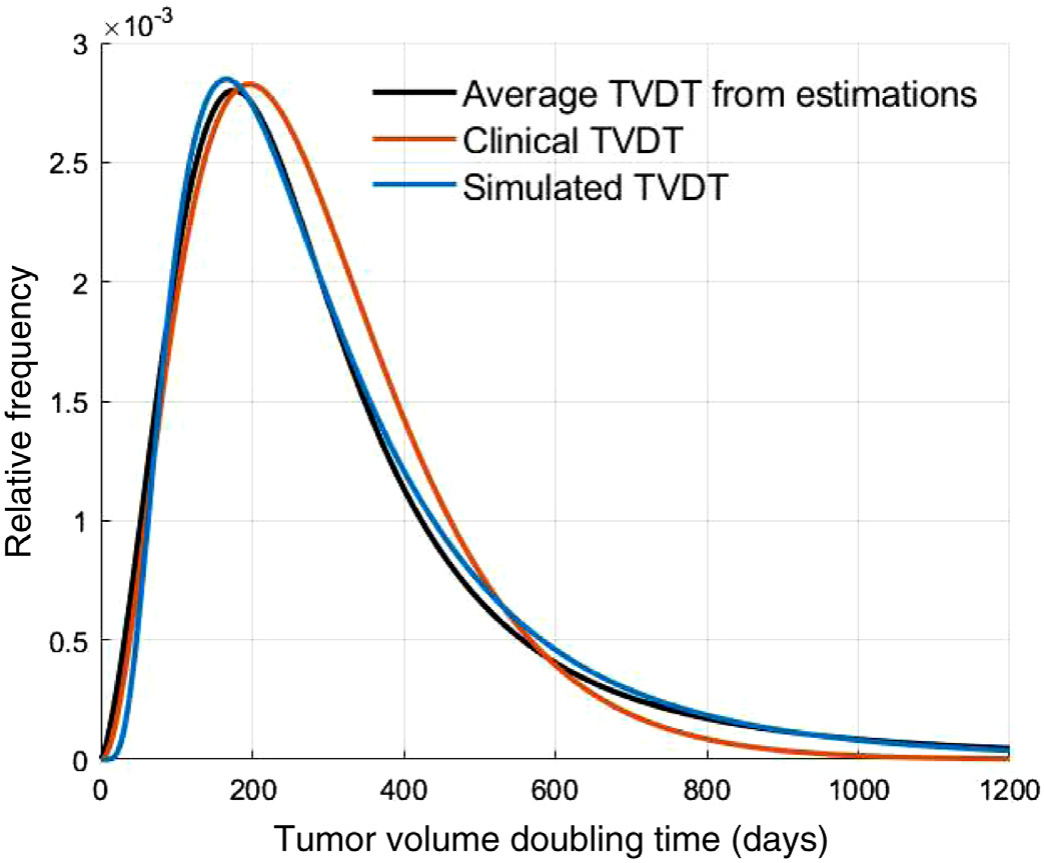
Probability distribution functions for the TVDTs. Shown are the average TVDT values estimated by the radiologist (black), the clinical TVDT values (red), and the TVDT values of the simulated cohort (blue).

Figure 8 compares the fitted distributions corresponding to the clinical TVDT, the TVDT from the simulated cohort, and the estimated TVDT values (average from the three measurement sessions). The results show an overlap of the TVDT distributions and similarities in their features (peak value, frequency, skewness, and tail).

The clinical data suggested a borderline normal distribution using the Shapiro–Wilk test (p=0.053). The TVDT values from the clinical fit, simulated cohort, and radiologist’s estimation did not follow a normal distribution (p<0.05). The borderline normal distribution for the clinical data was further investigated using the AD test. Results suggested that the clinical data was best described by a gamma distribution (Gamma dist.: p=0.94. Normal dist.: p=0.58). Consequently, the gamma distribution was used for further simulations of TVDT.

Due to non-normal distributions, we compared the TVDT data sets using the nonparametric two-sample Kolmogorov–Smirnov and Wilcoxon signed-rank tests.^38^^,^^40^ The p-values from the two statistical comparisons between the TVDT data sets are shown in Table 1. The median difference and interquartile range (IQR) of TVDT values from the simulated cohort and the radiologist’s estimation are listed in Table 2.

**Table 1:** Comparisons of the TVDT data sets using the two-sample Kolmogorov–Smirnov and Wilcoxon signed-rank test. Shown are p-values (using a significance level of 0.05).

TVDT data set statistical test	Clinical versus clinical fit	Clinical versus estimated (measurements 1, 2, and 3)	Simulated versus estimated (measurements 1, 2, and 3)	Simulated versus clinical fit
Two sample Kolmogorov–Smirnov	0.54	0.94, 0.94, and 0.94	0.54, 0.54, and 0.94	0.76
Wilcoxon signed-rank	0.94	0.69, 0.88, and 0.91	0.004, 0.006, and 0.040	0.64

**Table 2:** The median difference and IQR between TVDT values from the simulated cohort and radiologist’s estimates.

	TVDT values from the simulated cohort versus radiologist’s estimate		
	Measurement 1	Measurement 2	Measurement 3
Median difference (IQR) [days]	12 (21)	16 (27)	8 (29)

A significant difference in TVDT values was found between the simulated and the estimated TVDT, according to the Wilcoxon signed-rank test (Table 1).

The mean value of the median difference reported in Table 2 (12 days) corresponded to 4% of the clinically reported mean TVDT (282 days).

**Fig. 9:**
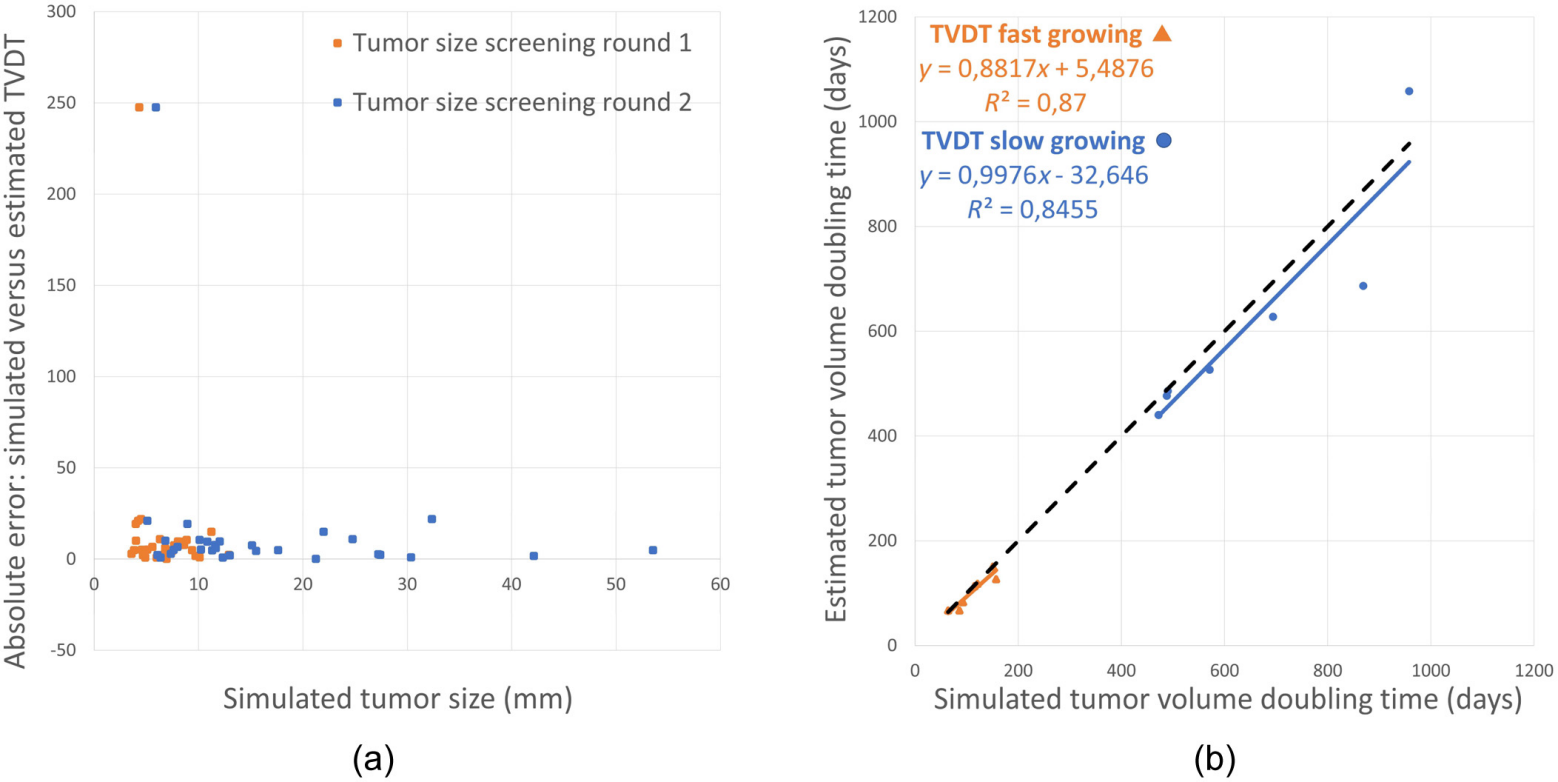
(a) The simulated (nominal) tumor sizes in the first and second screening round versus the absolute error between the simulated (nominal) and estimated TVDT value. (b) The estimated TVDT versus the simulated (nominal) TVDT for fast- and slow-growing tumors.

Figure 9(a) represents the absolute error between the simulated (nominal) and estimated TVDTs compared with the simulated (nominal) tumor size. The relationship between the estimated and simulated (nominal) TVDTs for fast- and slow-growing tumors is shown in Fig. 9(b). We found no significant difference when comparing the absolute relative error between the simulated (nominal) and estimated TVDTs for the fast-growing versus slow-growing tumors (p=0.89) according to the two-sample t-test.

The tumor sizes in Fig. 9(a) are distributed around an absolute error of 0% to 50% between the simulated (nominal) and estimated TVDTs. In Fig. 9(b), the R-squared value for fast- versus slow-growing tumors are in the same order of magnitude, and both regression lines are close to diagonal.

## 4 Discussion

This paper describes the development and evaluation of a preliminary method for tumor growth simulation using the OpenVCT pipeline. We have expanded OpenVCT to allow the simulation of clinical trials spanning over multiple screening rounds. This expansion enables novel simulation studies of the impact of new imaging modalities in screening programs, such as DBT, as well as the impact of tumor growth as an indicator for screening intervals or treatment. We anticipate that an analysis of the tumor growth rate may also help characterize and potentially identify aggressive malignant tumors.

We simulated tumors based on clinical results from the MBTST study.^13^ The sizes of the simulated tumors in the second screening round were selected to match the reported range of clinically detectable lesions on mammograms.^19^^,^^32^^–^^34^ Our method of simulating tumor growth suggested a high level of agreement between the simulated tumor size and the size estimated by the radiologist. This is evident in the average linear regression in Fig. 4, which is close to the diagonal. The Friedman test suggested no significant difference between the three repeated measurements (p>0.05).

Our simulation method succeeded in accurately representing the breast cancer growth rates reported previously in our clinical study.^19^ A substantial overlap was observed for the TVDT values in the clinical and simulated cohorts (Figs. 5 and 6). When fitted to probability distributions, the density of the distributions of the clinical, simulated, and estimated TVDT were skewed to the left, with a characteristic tail for larger TVDT values (Fig. 8). The distributions have approximately matching peak values (TVDT ∼200  days). Notably, the mean TVDT of our clinical fit [297 days (SD, 169 days)] differed slightly from the reported clinical value [282 days (SD, 164 days)]. Our clinical fit was created based on the histogram of clinical TVDT values published by Förnvik et al. and were within the ranges of other published studies.^14^^,^^16^^,^^19^^,^^41^^–^^43^

When comparing the analyzed TVDT data sets (Table 1), we observed no significant difference according to the two-sample Kolmogorov–Smirnov test (p>0.5). However, the Wilcoxon signed-rank test suggested a significant difference between the TVDT values from the simulated cohort and the radiologist’s estimates. The Kolmogorov–Smirnov and Wilcoxon signed-rank tests were used to assess whether the data follow the same distribution and to compare any difference in the mean ranks between data sets. The results of the Wilcoxon test imply the presence of a systematic difference between the simulated and estimated TVDT values. The effect of a systematic difference was assessed (Table 2). The observed difference between the simulated and the estimated TVDT data sets (8 to 16 days) may not be critical, as it corresponds to only ∼4% of the clinically reported mean TVDT [282 days, (SD 164 days)].

We measured the tumor size at two-time points and chose the interval to match the recommended screening intervals in Sweden.^31^ A few of the TVDT values sampled from our clinical fit resulted in palpable tumor sizes (>20  mm) in the second simulated screening round.^33^ In clinical cases, some of these tumors may have been detected before the second screening by the woman herself (interval cancers). As such, interval cancers are predominantly fast-growing tumors. In our study, we have not simulated interval cancers, which potentially limits the range of TVDT that we analyzed. However, the largest tumor size in our virtual population in the second screening round was 54 mm, which is still within the range of what is seen clinically.^44^

The accuracy of the estimated TVDTs did not appear to be dependent on the tumor size [Fig. 9(a)]. Future work will include a larger sample of simulated tumors and test the effect of tumor size on the accuracy and reproducibility. Moreover, we found no significant difference in the accuracy of TVDT estimations between the fast-growing tumors versus slow-growing tumors [Fig. 9(b)]. Most likely it is not the tumor size or tumor growth rate affecting the manual estimation of TVDT, but the location and shape of the tumor. A tumor masked by the anatomical structures of the surrounding breast parenchyma should yield higher errors between the estimated and simulated TVDT due to difficulties defining the tumor edges. In this study, the tumors were estimated to be spherical masses. However, despite a simplistic tumor shape, the surrounding simulated breast anatomy sometimes masked the tumor margin. In clinical settings, most radiologists only report the bulk size of the tumor. Therefore, we believe that we achieved an acceptable degree of difficulty in measuring the simulated tumors for this preliminary study. Our group has ongoing projects where we are exploring simulations of irregularly shaped tumors as well.^45^ This would allow for a more plausible representation of the tumor shape and its temporal changes, which likely affect both size measurements and TVDT estimations.

The effect of breast density is also an important consideration because it could introduce false positives. Another limitation is the absence of temporal change in the breast parenchyma between the simulated screening rounds, which may impact the TVDT estimation. Future studies should focus on formulating a robust method for examining the local tissue density compared with estimations of tumor size and TVDT. Furthermore, we intend to analyze the effect of molecular phenotypes on the tumor growth rate by using clinically available data. Two previous studies at our institution have related tumor growth to histopathological characteristics and molecular subtypes.^19^^,^^46^ The growth analysis of tumors from the MBTST^13^ (101 cases with circumscribed or spiculated masses, with at least two consecutive mammograms^46^), is currently being performed by our group.^47^ We intend to use these studies to enable VCTs with different simulated phenotypes in order to investigate the use of growth rate in clinical decision-making about screening or treatment.

All lesions were simulated as masses in this study; radiographic masses were the predominant type of lesions detected in MBTST (110 out of 139). To select patient age, we looked at the age distribution at cancer detection from the MBTST.^13^ We applied the same age distribution to our study. The intention of assigning patient age to the virtual cohort was to achieve a realistic distribution of women being screened at 18- or 24-month intervals (women aged <55  years are screened at 18-month intervals at our institution). The interval affects the “end size” (the size at detection) of the tumor. Therefore, a realistic distribution of the intervals should also yield realistic tumor size distributions in the simulated cohort. This approach of selecting tumor size resulted in simulated tumors within the clinically plausible size range.^44^ Moreover, we set the interval between the two screening sessions to be strictly 18 or 24 months, depending on the patient’s age. However, there is potential to include a random variation within the screening intervals, to further mimic a realistic screening environment. Using available clinical data, we could study this pattern and introduce it as an additional variable for future VCTs.

Only one radiologist participated in this study. However, for the scope of this preliminary study, we found it to be sufficient to only have one radiologist and instead focus on the intrareader variability. For our future studies, where we will most likely add more advanced tumor shapes, we will include multiple radiologists and explore the use of AI to estimate lesion sizes. Moreover, which modality is preferable for estimating TVDTs is still unclear. Clinical TVDTs for breast tumors have been previously reported based on breast cancer data.^14^^,^^19^^,^^32^^,^^48^ Förnvik et al.^49^ showed that DBT was significantly superior to DM when it came to determining breast tumor size. Other studies have also shown that ultrasound and MRI are suitable when estimating tumor growth.^16^^,^^50^ The advantage of ultrasound and MRI is that several tumor size measurements can be made in the same woman, as there is no risk for harm by ionizing radiation. However, TVDT estimates from ultrasound and MRI may not accurately represent the TVDTs from a DM or DBT screening cohort. Finally, our method for simulating tumor growth has the potential to aid the clinical discussion regarding personalized screening based on tumor aggressiveness and, therefore, it is advantageous if the TVDT is derived from a screening population.

## 5 Conclusions

This study presents a preliminary method for simulating tumor growth that enables VCTs of breast cancer screening over multiple screening rounds. Our evaluation of the method suggested no significant difference between the TVDTs manually estimated from simulated images by a radiologist [340 days (SD, 287 days)] and clinical values [282 days (SD, 164 days)].
